# *Astragalus* polysaccharides alleviates lipopolysaccharides-induced inflammatory lung injury by altering intestinal microbiota in mice

**DOI:** 10.3389/fmicb.2022.1033875

**Published:** 2022-10-25

**Authors:** Ke Ming, Shen Zhuang, Ning Ma, Sha Nan, Qiuhua Li, Mingxing Ding, Yi Ding

**Affiliations:** College of Veterinary Medicine, Huazhong Agricultural University, Wuhan, China

**Keywords:** inflammatory lung injury, *Astragalus* polysaccharides, intestinal microbiota, short chain fatty acids, anti-inflammation

## Abstract

Inflammatory lung injury is a common respiratory disease with limited therapeutic effects. Increasing opinions approved that prevention is more important than drug treatment for inflammatory lung injury. *Astragalus* polysaccharides (APS) has multiple bioactivities including anti-inflammation and immunoregulation. However, its preventive effects on inflammatory lung injury remain unclear. In this study, mice were pretreated with APS *via* intragastric gavage and then were intratracheally instilled with lipopolysaccharides (LPS) to determine the role of APS in preventing lung injury. The results showed that APS pre-treatment improved the pathological changes of lung tissues, reduced the neutrophils infiltration, and inhibited the LPS-induced inflammation. Increasing evidence confirmed the close relationship between intestinal microbiota and lung inflammatory response. 16S rRNA analysis showed that APS treatment changed the microbiota composition in colon, increased the abundance of short-chain fatty acids (SCFAs)-producing genus such as *Oscillospira*, *Akkermansia*, and *Coprococcus*. Also, APS treatment significantly increased the serum concentrations of SCFAs including butyrate and propionate, and their anti-inflammation effects were demonstrated on mice primary alveolar macrophages. Our data confirmed the preventive effects of APS on LPS-induced lung injury, which were partly contributed by the alteration of intestinal microbiota composition and the resulting increase of serum SCFAs.

## Introduction

The lung is directly exposed to the external environment and therefore susceptible to pathogens or harmful substances in the air. Inflammatory lung injury from a variety of causes remains a heavy burden that affects people and livestock, and results in more than 4 million deaths worldwide, as well as huge economic losses in hoggery (Ferkol and Schraufnagel, 2014; Liu et al., 2022). Inflammatory lung injury is characterized by the damage of alveolar-capillary barrier, inflammatory cells infiltration, and pulmonary edema (Matthay et al., 2012; Bhattacharya and Matthay, 2013). Lipopolysaccharides (LPS) is an endotoxin from Gram-negative bacteria and is of particular significance in immunology and microbiology. LPS exposure is one of the major reasons for the development of inflammatory lung injury in human and animals. Current medication strategy for lung injury includes mechanical ventilation, antibiotics, and hormone drugs (Levitt and Matthay, 2012). However, the available treatments are limited in quantity and impact, especially for the acute individuals. Furthermore, the overuse of drugs results in not only various side effects, but also drug residues, which are harmful to environment and host health. Therefore, increasing opinions approved that the prevention of inflammatory lung injury is more important than drug treatment (Litell et al., 2011).

Natural products from herb or food have attracted people’s attention due to their wide bioactivities and low toxic and side effects, and have been used in functional additives (Kourkoutas et al., 2015). *Astragalus* polysaccharides (APS) is the primary bioactive ingredient isolated from *Astragalus membranaceus*, a representative Traditional Chinese medicine. Studies have found that APS possesses ideal effects in suppressing inflammation and regulating immune system on cells and animals (Lu et al., 2013; Wang et al., 2013; Zhou et al., 2017; Li et al., 2022). What is more, APS was reported to be synergistic, when combined with matrine, in relieving lung injury associated with inflammatory bowel disease (IBD), and also has been reported to protect lung from developing to pulmonary arterial hypertension (Yuan et al., 2017; Yan et al., 2020). Even so, the role and mechanisms of APS on lung health is still largely unknown.

Intestinal microbiota is a collection of microorganisms that lives in host’s body, among which friendly and potential pathogenic bacteria are included. Microbial dysbiosis leads to disorders such as IBD and asthma (Ferreira et al., 2014). Increasing studies have shown that intestinal microbiota regulates host immunity in a choreographed way, and the cross talk between them extends beyond the gut environment and affects the immunity of distal tissues, including lung (Ichinohe et al., 2011; Brown and Clarke, 2017; Budden et al., 2017). For instance, diverse gut microbiota protect murine against mortality induced by pneumococcal pneumonia (Schuijt et al., 2016). Even for neonates, gut commensal bacteria has played its role in lung mucosal immunity (Gray et al., 2017). Those studies demonstrated the close association between gut microbiota and lung health. Intestinal microbiota affects host health mostly by fermenting dietary fibers and producing short chain fatty acids (SCFAs; Cummings et al., 1987; Macfarlane and Macfarlane, 2012). SCFAs regulate immune cells to enhance host immunity and inhibit the deterioration of inflammation (Yao et al., 2022).

The composition of intestinal microbiota is influenced by many factors, including diet, age, illness, and medication (Purchiaroni et al., 2013). It has been demonstrated that supplementation of APS in diet protected mice from nonalcoholic fatty liver disease, and the underlying mechanisms depended on the remodel of gut microbiota (Hong et al., 2021). Moreover, APS can alter the gut microbiota to improve the osteoporosis (Liu et al., 2020). Based on these findings, here we investigated the potential benefits of APS on LPS-induced inflammatory lung injury and further explored the role of intestinal microbiota in this process.

## Materials and methods

### The preparation of APS and monosaccharides composition analysis

The APS was extracted from *Astragalus membranaceus* by boiling water extraction, and then the supernatant was deproteinized using Sevag reagent (Sevag et al., 1938). After removing Sevag reagent, the liquid was mixed with 95% ethanol to a final concentration of 80% (v/v). Twenty-four hours later, the precipitation was dried to get the crude APS. The crude polysaccharides were eluted by water using DEAE-cellulose column to obtain the purified APS.

The monosaccharides composition of APS was analyzed with High-Performance Liquid Chromatography (HPLC; Dionex U3000, ThermoFisher) equipped with ZORBAX EclipseXDB-C18 (Agilent) column. The mobile phase was acetonitrile and phosphate-buffered saline (12 g/L of KH_2_PO4, 2 M NaOH, and adjust the pH to 6.8) in a proportion of 17:83, the flow rate was 0.8 ml/min and the wavelength was 250 nm. Five point 2 mg of the purified APS was dissolved in trifluoroacetic acid (TFA) solution at 121°C for 2 h and dried with termovap sample concentrator. Then methanol was used to wash the sample till the TFA was completely removed. The sample and standard monosaccharides were reacted with PMP reagent (1-phenyl-3-methyl-5-pyrazolone) before HPLC analysis.

### Animals and treatment

Male C57BL/6 mice aged 5 weeks were purchased from Laboratory Animal Center of Huazhong Agricultural University. Mice were housed under even temperature (22 ± 2°C) and 12 h light/12 h dark cycle. After a week of acclimatization, mice were randomly divided into four groups, control group, LPS group, APS + LPS group, and APS group, respectively. Mice in control group and LPS group were administered with water by intragastric gavage for consecutive 14 days while mice in APS group and APS + LPS group were administered 200 mg/kg of APS (dissolved in water) in the same way. At the 15th day, all the mice were anesthetized with 1% of pentobarbital sodium and intratracheally instilled with PBS (for control group and APS group) or LPS (2 mg/kg, dissolved in PBS, for LPS group and APS + LPS group, Sigma, St Louis, MO, United States).

### Wet/dry ratio of lung tissue

Lung tissues were separated from thoracic cavity, and the trachea and esophagus were removed. The lungs were numbered, and the wet weights were measured. Subsequently, the tissues were placed into oven at 60°C for 48 h to desiccate followed by weighting the dry lungs. The wet/dry (W/D) ratio of each lung tissue was calculated.

### H&E staining

The lung tissues were fixed overnight and embedded in paraffin. Then, the slides were dehydrated using ethanol and stained with hematoxylin and eosin (H&E). The slides were observed under optical microscope (Olympus Shinjuku-ku, Tokyo, Japan).

### Bronchoalveolar lavage fluid and primary alveolar macrophages

Bronchoalveolar lavage fluid (BALF) was obtained by inserting 24-gage catheter into trachea and flushing with 1 ml of PBS for three times. The BALF was centrifuged at 400 × g for 10 min to remove liquid, and the cells pellets were collected for cell counting or cell culture. The cells attached to the cell plate were alveolar macrophages. Total protein concentration in cell-free BALF were measured by BCA protein assay kit (Biosharp, cat. no. BL521A).

### RNA isolation and real-time qPCR assay

Total RNA from lung tissues were isolated using RNA isolater Total RNA Extraction Reagent (Vazyme Biotech, cat. no. R401-01) and then were reversely transcribed into complementary DNA using ABScript III RT Master Mix (ABclonal Technology, cat. no. RK20429). Universal SYBR Green Fast qPCR Mix (ABclonal Technology, cat. no. RK21203) and specific primers were mixed with cDNA for qPCR assay. Two delta delta Ct method was used to analyze the relative expression levels of certain genes in lung homogenates. The primer sequences were shown in Table 1. The qPCR values were processed by TBtools software (version 1.0), in which the log scale to base 2 and column scale were selected and heatmap was drawn.

**Table 1:** The specific primers for qPCR.

Primers	Sequence (5′ to 3′)	References
mice Il-1β F	GAAATGCCACCTTTTGACAGTG	Zhou et al. (2020)
mice Il-1β R	TGGATGCTCTCATCAGGACAG	
mice Il-6\u00B0F	CTGCAAGAGACTTCCATCCAG	
mice Il-6 R	AGTGGTATAGACAGGTCTGTTGG	
mice Tnf-α F	CCCTCACACTCAGATCATCTTCT	
mice Tnf-α R	GCTACGACGTGGGCTACAG	
mice Cxcl1 F	CTGGGATTCACCTCAAGAACATC	
mice Cxcl1 R	CAGGGTCAAGGCAAGCCTC	
mice Cd86 F	CAGACTCCTGTAGACGTGTTC	
mice Cd86 R	GTCCCATTGAAATAAGCTTGCG	
mice Mrc1 F	CCACGGATGACCTGTGCTCGAG	
mice Mrc1 R	ACACCAGAGCCATCCGTCCGA	
mice Ccl2 F	TACAAGAGGATCACCAGCAGC	Lu et al. (2017)
mice Ccl2 R	ACCTTAGGGCAGATGCAGTT	
mice Icam-1 F	CACCCCGCAGGTCCAAT	Qian et al. (2022)
mice Icam-1 R	CAGAGCGGCAGAGCAAAAG	
mice Vcam-1 F	GACTCCATGGCCCTCACTTG	
mice Vcam-1 R	GCGTTTAGTGGGCTGTCTATCTG	
mice Actin F	GTGACGTTGACATCCGTAAAGA	Chang et al. (2021)
mice Actin R	GCCGGACTCATCGTACTCC	

### ELISA analysis

The cytokine concentrations in lung tissue and serum were measured by ELISA assay. The lung tissues were homogenized in cold PBS and centrifuged to collect supernatant, in which the total protein levels were quantified by BCA assay. The lung homogenates and serum were then subjected to ELISA kit (Enzyme-linked Biotechnology) to detect the concentration of IL-1β (cat. no. ml301814), IL6 (cat. no. ml063159), and TNF-α (cat. no. ml002095).

### Flow cytometry

Flow cytometry was performed to identify alveolar cell subpopulations. The cells collected in BALF was stained with CD11b-PerCP Cy5.5 (BD Pharmingen, 561114), Ly-6G-FITC (Thermo Fisher Scientific, 11–9668-82), F4/80-eFlour 450 (Thermo Fisher Scientific, 48–4801-80), CD206-APC (Thermo Fisher Scientific, 17–2061-80), or CD86-PE (Thermo Fisher Scientific, 12–0862-81; all diluted 1:200) according to the manual. Samples were submitted to Cytoflex-LX Flow Cytometer (Beckman), and data were analyzed by using FlowJo (BD Bioscience).

### Western blot

The protein samples from lung tissues were prepared, and BCA method was used to detect the protein concentration in each sample. Twenty micrograms of total protein were loaded into wells of 10% SDS-PAGE gel. After being separated by electrophoresis, proteins were transferred to PVDF membrane (Millipore) and then blocked using 5% milk. Then, the membrane was incubated with NF-kB p65/RelA Rabbit pAb (ABclonal Technology, A2547, diluted 1:1,000), Phospho-NF-kB p65/RelA-S536 Rabbit pAb (ABclonal Technology, AP0475, diluted 1:1,000), and β-Actin Rabbit pAb (ABclonal Technology, AC006, diluted 1:2,000) overnight at 4°C. Appropriated secondary antibody was incubated with membrane at room temperature for 2 h, and then protein bands were visualized using Image-Pro Plus 6.0 software (Media Cybernetics), and grey values were measured by ImageJ software.

### 16S rRNA analysis

The colon samples were immediately frozen and stored at −80°C, and the microbiota compositions were analyzed *via* 16S rRNA analysis. The total DNA was extracted and then PCR amplification (ABI 2720, United States) of the 16S rRNA genes V3-V4 region were performed using specific primers (338F: 5′-ACTCCTACGGGAGGCAGCA-3′; 806R: 5′-GGACTACHVGGGTWTCTAAT-3′). The products of PCR were then sequenced by Novaseq-PE250 platform at Personal Biotechnology Co., Ltd. (Shanghai, China). The sequence data were analyzed using QIIME2 and R package.

In data analysis, alpha-diversity indices were used to evaluate the intestinal microbiota diversity and abundance. Principal coordinate analysis (PCoA) based on Bray-Curtis distance was used to compare the microbiota composition of each group. The relative species abundances were shown at the phylum and genus levels among groups.

### Quantification of SCFAs

The serum concentrations of SCFAs determined by Gas Chromatography–Mass Spectrometry (GC–MS). Standard SCFAs (Sigma-Aldrich, United States) were diluted in methyl tert-butyl ether (MTBE). 100 μl of serum samples were mixed with 0.5 ml of MTBE (with internal standard) solution followed by incubating for 3 min. The mixtures were then ultrasonicated for 5 min, and centrifuged at 12,000 r/min for 10 min. The supernatant was collected and used for GC–MS/MS analysis. Gas chromatograph (Agilent 7890B) coupled to a mass spectrometer (7000D) with a DB-5MS column (30 m length × 0.25 mm i.d. × 0.25 μm film thickness, J&W Scientific, United States) was used for GC–MS/MS analysis. Helium was used as the carrier gas at a flow rate of 1.2 ml/min. Injections in splitless mode were made with an intection volume of 2 μl. The oven temperature was kept at 90°C for 1 min, raised to 100°C at a rate of 25°C/min, raised to 150°C at a rate of 20°C/min, hold for 0.6 min, raised to 200°C at a rate of 25°C/min, and held for 0.5 min after running for 3 min. All samples were analyzed in multiple reaction monitoring mode. The temperatures of inlet and transfer line were 200 and 230°C, respectively.

### Statistical analysis

All data were presented as the mean ± SD. Significant differences among groups were analyzed by one-way analysis of variance (ANOVA) and *t*-tests using GraphPad Prism 8 software. The heatmap was drawn by TBtools software following data normalization by using log scale and column scale.

## Results

### Monosaccharides composition of APS

**Figure 1:**
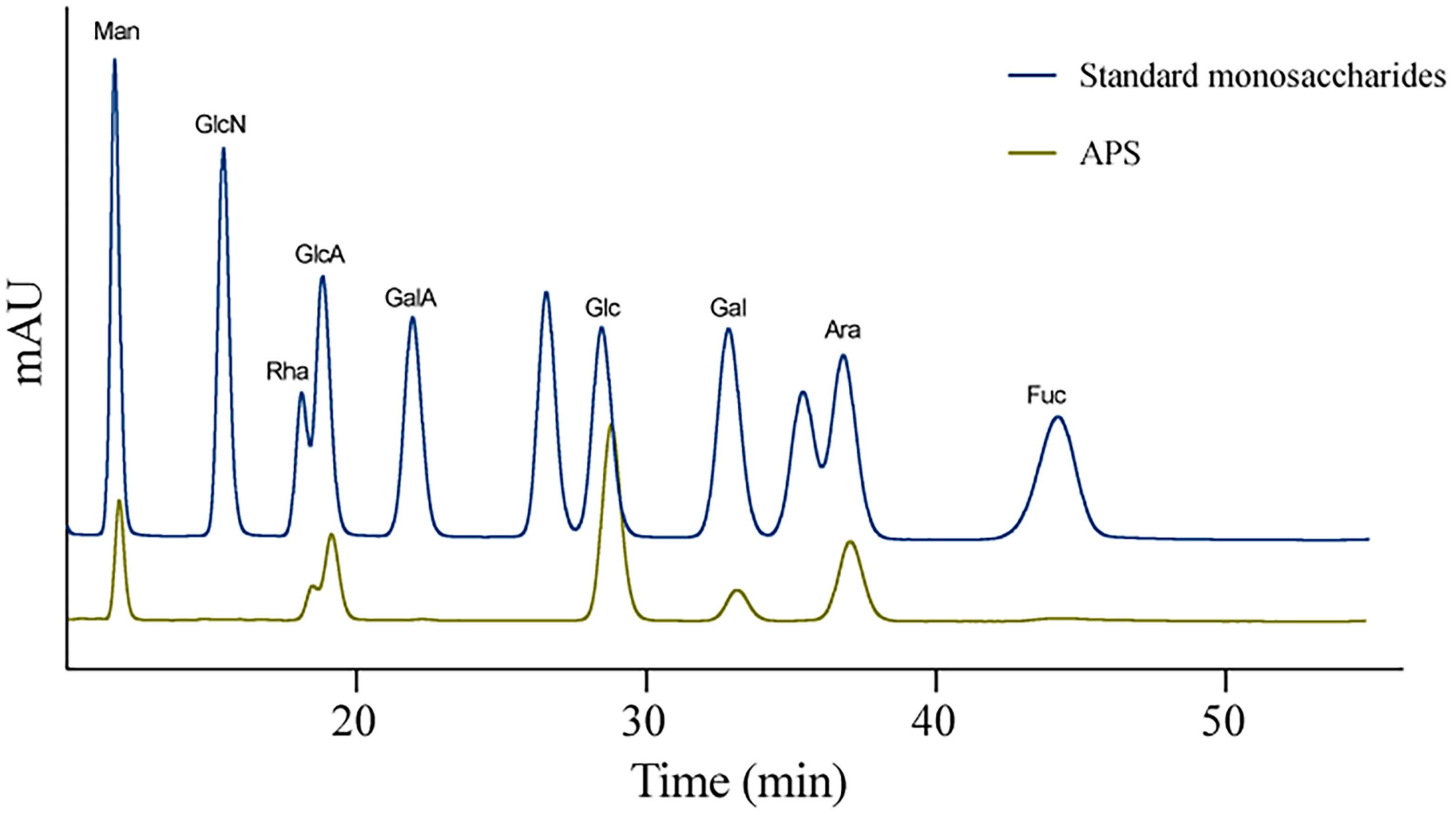
The monosaccharides composition of *Astragalus* polysaccharides (APS).

High-Performance Liquid Chromatography was used to detect the monosaccharides composition of APS according to the standard monosaccharide samples. Results showed that the APS used in this study was composed of mannose (Man, 8.922%), rhamnose (Rha, 10.255%), glucuronic acid (GlcA, 12.552%), glucose (Glc, 39.283%), galactose (Gal, 5.418%), and arabinose (Ara, 23.570%) (Figure 1).

### APS pre-treatment ameliorates inflammatory lung injury

**Figure 2:**
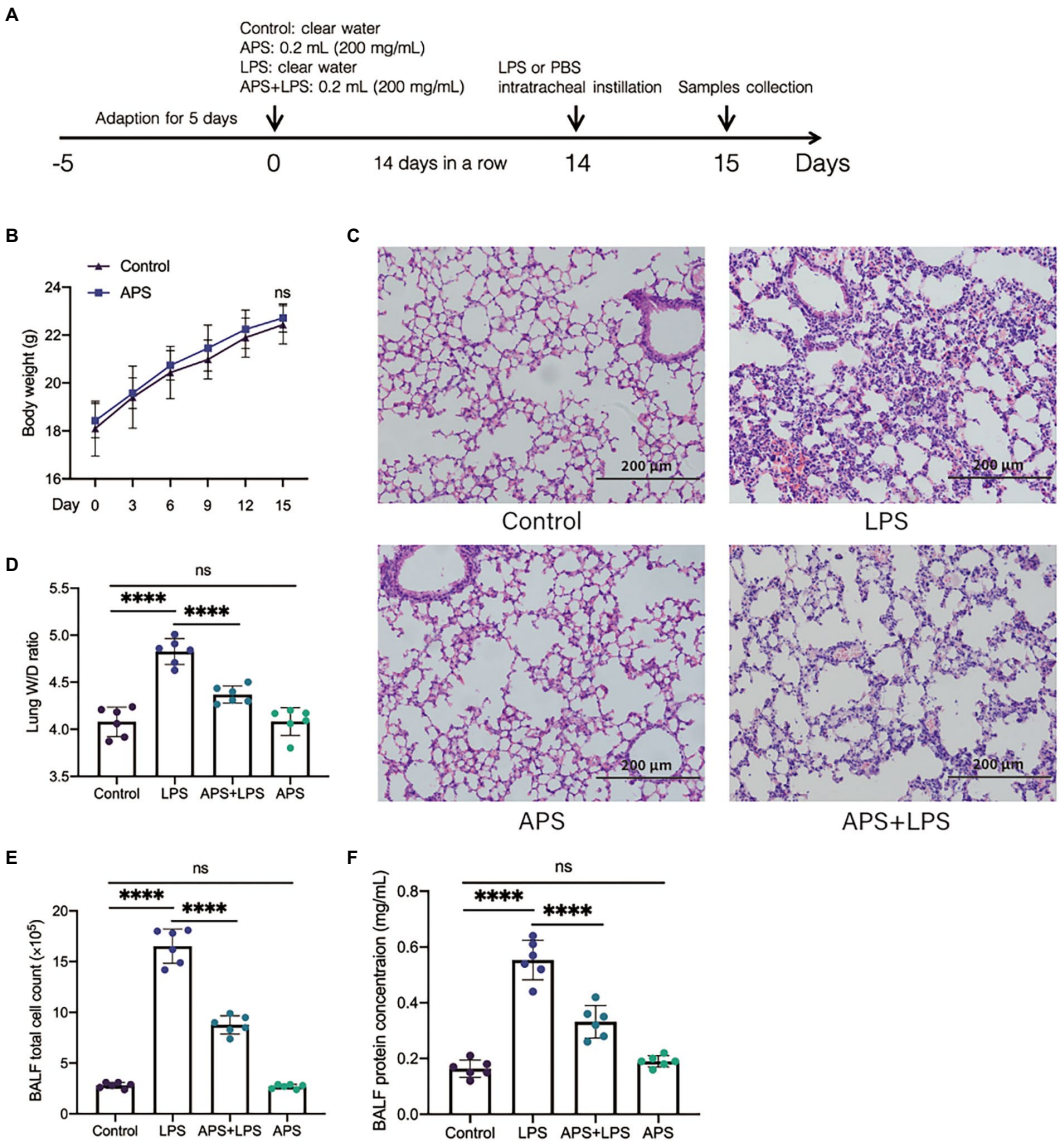
APS pre-treatment alleviated the inflammatory lung injury following lipopolysaccharides (LPS) exposure. **(A)** The schematic of the experimental design. Twenty-four hours post LPS instillation, samples were collected. **(B)** The body weight of mice with or without APS treatment before LPS exposure. **(C)** Representative pictures of lung tissues with hematoxylin and eosin (H&E) staining. **(D)** The ratio of lung wet weight and dry weight with *n* = 6 sample per group. **(E)** The number of total cells in bronchoalveolar lavage fluid (BALF) with *n* = 6 sample per group. **(F)** The total protein concentration in BALF was measured by BCA analysis with *n* = 6 sample per group. Statistical significance was determined by one-way ANOVA using GraphPad Prism 8 software. ^****^p < 0.0001.

To confirm the protective role of APS on mice lung tissue, the mice were treated with APS solution for consecutive 2 weeks and then inflammatory lung injury mice models were established by LPS (Figure 2A). Data showed that APS treatment did not affect the body weight of mice (Figure 2B). Intratracheal instillation of LPS resulted in notably pathologic changes including destroyed alveolar structure, thickened alveolar wall and infiltration of inflammatory cells and red blood cells. However, in APS pre-treatment group, the pathologic changes induced by LPS were less severe, as more alveoli and less inflammatory cells were observed (Figure 2C). We calculated the W/D ratio of the lungs to evaluate their edema. Results showed that LPS instillation markedly increased the W/D ratio, suggesting the development of lung edema. As expected, APS pre-treatment prevented the increase of W/D ratio and mitigated the lung edema (Figure 2D).

We also collected the BALF and measured the total cell count and total protein concentration. The results showed that LPS treatment increased the quantity of total cells as well as the concentration of total protein in BALF, while these two indices were significantly reduced in APS pre-treatment (Figures 2E,F). This further proved that APS pre-treatment alleviated the inflammatory lung injury induced by LPS.

### APS pre-treatment inhibits LPS-induced inflammatory response in lung

**Figure 3:**
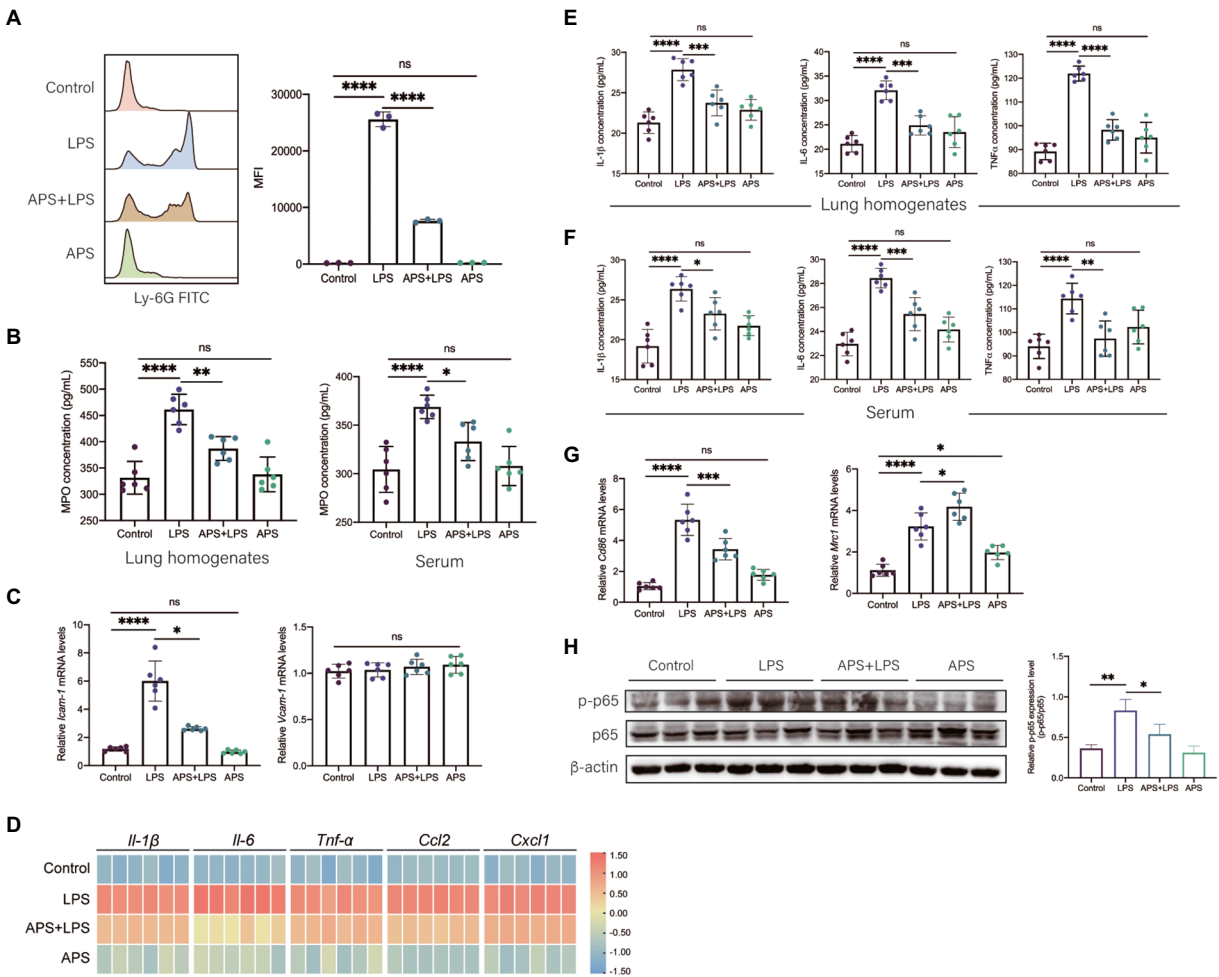
APS inhibited the inflammatory response induced by LPS. **(A)** The number of neutrophils in BALF was measured by flow cytometry. Representative flow cytometry histograms showing mean fluorescence intensity (MFI). MFI values were quantified *n* = 3 samples per group. **(B)** The myeloperoxidase (MPO) concentrations in lung homogenates and serum were measured by ELISA with *n* = 6 sample per group. **(C)** The mRNA expression levels of Icam-1 and Vcam-1 with *n* = 6 sample per group. **(D)** The heatmap represents the mRNA expression levels of pro-inflammatory genes in lung tissues with *n* = 6 samples per group. The relative expression data were normalized by using log scale and column scale and the heatmap was drawn in TBtools software. **(E)** The concentration of representative pro-inflammatory cytokines (IL-1β, IL-6, and TNF-α) in lung homogenates with *n* = 6 samples per group. **(F)** The concentration of representative pro-inflammatory cytokines (IL-1β, IL-6, and TNF-α) in serum with *n* = 6 samples per group. **(G)** The mRNA expression levels of M1 macrophages marker (Cd86) and M2 macrophages marker (Mrc1) in lung tissues with *n* = 6 samples per group. **(H)** The relative p-p65 expression level in lung tissue was presented by the ratio of p-p65 to p65 with *n* = 3 samples per group. High expression of p-p65 represents the activation of NF-κB signals in lung. Statistical significance was determined by one-way ANOVA using GraphPad Prism 8 software. ^*^p < 0.05, ^**^p < 0.01, ^***^p < 0.001, ^****^p < 0.0001.

Lipopolysaccharides activates immune cells to overproduce inflammatory cytokines chemokines and lead to severe inflammation in tissues. Neutrophils are of importance in the onset of tissue inflammatory injury. Thus, we firstly evaluated the role of APS on the neutrophils infiltration. Flow cytometry analysis showed that abundant neutrophils were detected in lungs with inflammatory injury, and APS pre-treatment significantly reduced the number of neutrophils (Figure 3A). The concentration of myeloperoxidase (MPO) in lung homogenates and serum were measured by ELISA. Results showed that LPS induced remarkable increase of MPO concentration in both lung homogenates and serum. Consistent with flow cytometry results, APS pre-treatment downregulated the MPO concentration (Figure 3B). Moreover, we also detected the relative mRNA levels of intercellular adhesion molecule-1 (ICAM-1) and vascular cell adhesion molecule-1 (VCAM-1), which were responsible for the adhesion of neutrophils to vascular endothelium (Wiesolek et al., 2020). Results showed that LPS treatment significantly increased the expression of *Icam-1* in mice lung but had no effect on the expression of *Vcam-1*. APS pre-treatment led to less *Icam-1* expression but did not change the expression of *Vcam-1* either (Figure 3C). These results further confirmed that APS prevented the neutrophils infiltration in lung inflammatory response.

We then detected the expression of various pro-inflammatory markers. Twenty-four hours post LPS exposure, qPCR was used to analyze the relative mRNA levels. The results showed that LPS treatment greatly increased the relative expressions of *Il-1β*, *Il-6*, *Tnf-α*, *Ccl2*, and *Cxcl1*, which were significantly reduced in APS pre-treatment group (Figure 3D). Same trends were also observed by ELISA analysis, the concentrations of IL-1β, IL-6, and TNF-α in both lung homogenates and serum remarkably increased in LPS treatment group yet decreased when mice were pretreated with APS (Figures 3E,F).

We also observed the phenotype of alveolar macrophages (AMs), which is another type of immune cells responsible for the secretion of cytokines in lung. Pro-inflammatory macrophages express high CD86 while anti-inflammatory macrophages express high mannose receptor (MRC1, also known as CD206). After 24 h of LPS stimulation, AMs were collected from BALF and qPCR was conducted to detect the mRNA expression of *Cd86* and *Mrc1*. We observed that LPS treatment significantly increased the expression of *Cd86*, and APS pre-treatment reduced its expression (Figure 3G). LPS treatment also induced the increase of *Mrc1*, this suggested adaptive anti-inflammatory response of AMs was elicited by LPS. APS combined with LPS further increased the *Mrc1* expression (Figure 3G). This indicated that APS pre-treatment instructed the transition of macrophages from pro-inflammatory phenotype to anti-inflammatory phenotype.

Lipopolysaccharides can be recognized by toll like receptor 4 (TLR4) that located on the surface of several immune cells, and then the downstream NFκB signaling pathway was activated to produce inflammatory cytokines (Lu et al., 2008). The activation of NFκB is a crucial step to produce inflammatory cytokines. We observed the increase of the phosphorylation of p65 protein in lung following LPS treatment, while the phosphorylated p65 expression level in APS pre-treatment group was significantly decreased, suggesting the inhibition of NFκB signaling pathway (Figure 3H).

### APS pre-treatment changes the intestinal microbiota of mice

**Figure 4:**
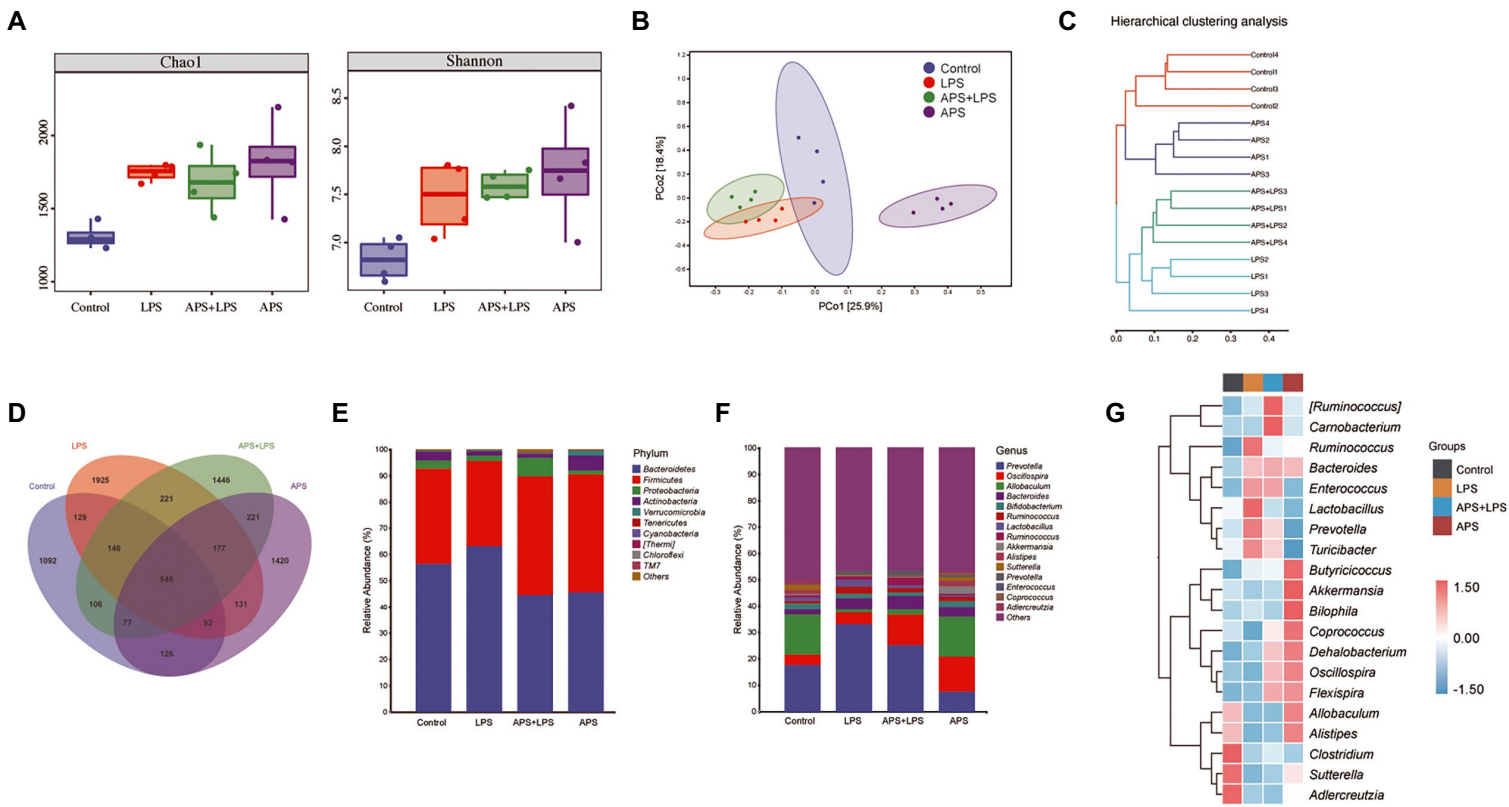
APS pre-treatment altered the intestinal microbiota composition. **(A)** Chao1 indices and Shannon indices of microbiota in colon with *n* = 4 samples per group. **(B)** Principal coordinate analysis (PCoA) represented the β-diversity of microbiota composition with *n* = 4 samples per group. **(C)** Cluster tree based on unweighted pair-group method with arithmetic mean (UPGMA) analysis. **(D)** The Venn diagram showed the overlaps of operational taxonomic units (OTUs). **(E)** The relative abundance of the top 10 phyla among the groups with *n* = 4 samples per group. **(F)** The relative abundance of the top 15 genus among the groups with *n* = 4 samples per group. **(G)** Heatmap represented the relative abundance changes of the top 20 genus among groups with *n* = 4 samples per group.

To explore the role of APS on mice intestinal microenvironment, the microbiota of colon mucosa was analyzed by 16S rRNA sequencing. Alpha-diversity based on Chao1 index and Shannon index was used to evaluate the community richness and diversity (Figure 4A). Results showed that compared to control group, Chao1 index and Shannon index values were increased in all other three groups. Principal coordinates analysis (PCoA) was used to evaluate the overall difference in microbiota classification. Samples in same group clustered together and were separated from other groups, suggesting the microbiota composition was affected by LPS and APS treatment (Figure 4B). The unweighted pair-group method with arithmetic mean (UPGMA) analysis showed that samples within same group were clustered together and distinguished with each other, confirmed that those treatment significantly altered the microbiota composition (Figure 4C). The Venn diagram illustrated the overlaps of Operational taxonomic units (OTUs) profiling and showed that a total of 545 OTUs among four groups (Figure 4D). Besides, the total number of microorganisms was increased in LPS treatment groups and decreased with APS pre-treatment. What is more, APS alone treatment also increased the number of microorganisms (Figure 4D).

Then, we analyzed the relative abundance of dominant taxa. At phylum level, Firmicutes and Bacteroidetes, constituted the principal part of microbiome community. Lung injury mice had higher level of Bacteroidetes but lower level of Firmicutes. On the contrary, mice pretreated with APS had higher Firmicutes but lower Bacteroidetes (Figure 4E). At genus level, LPS treatment increased the proportion of *Prevotella*. Compared with control group and lung injury group, APS pretreatment significantly reduced the *Prevotella* proportion and increased the *Oscillospira* proportion. LPS treatment also reduced the proportion of *Allobaculum*, whereas APS pre-treatment did not alter this reduction (Figure 4F). Besides, APS pre-treatment also increased the proportion of *Akkermansia* and *Coprococcus*, inspite of their relative low level in the whole genus (Figures 4F,G).

### APS pre-treatment inhibits lung inflammatory response *via* increasing the serum concentration of SCFAs

**Figure 5:**
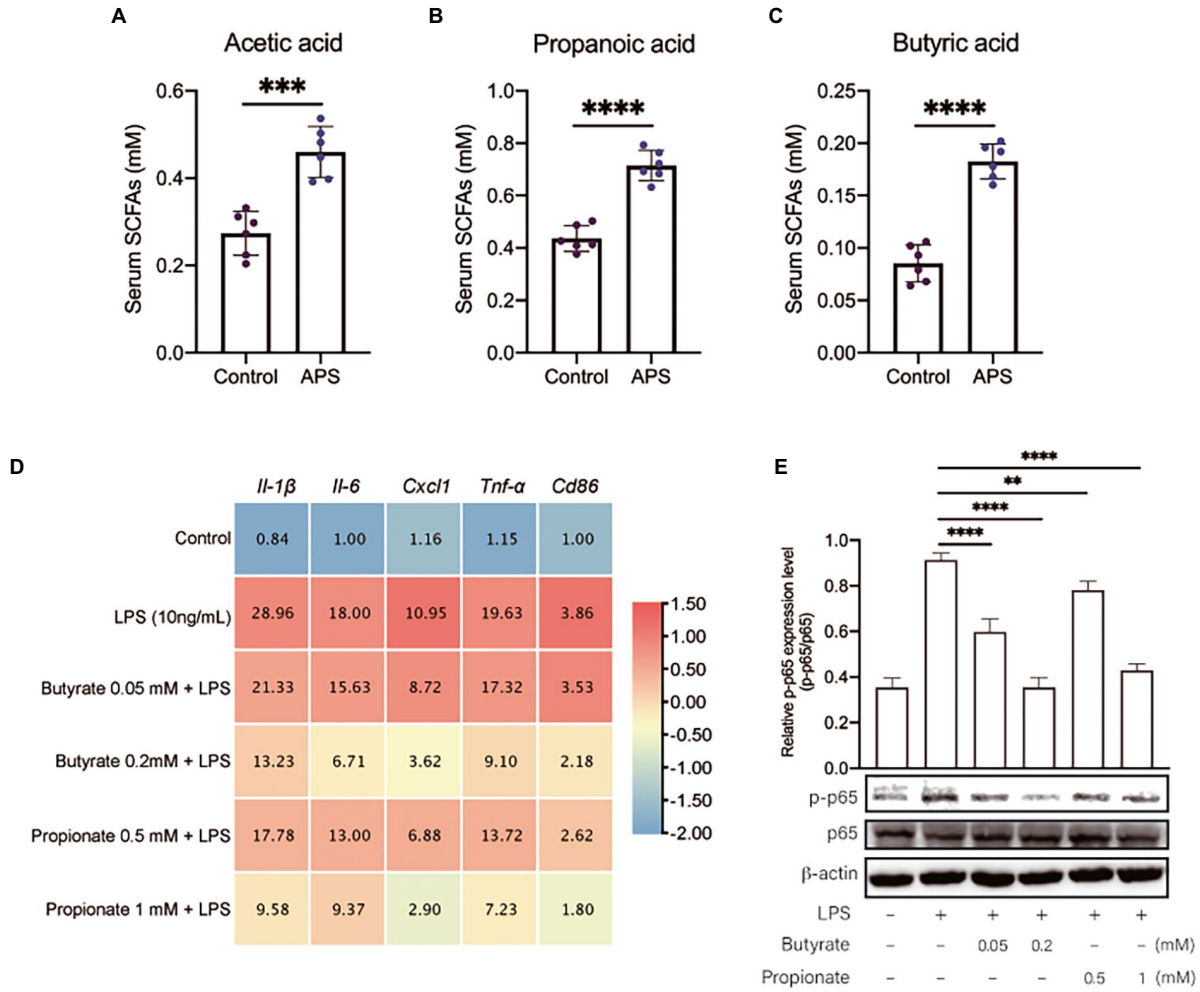
APS pre-treatment inhibits lung inflammatory response *via* increasing the serum concentration of SCFAs. **(A)** The serum concentration of Acetic acid with *n* = 6 samples per group. **(B)** The serum concentration of Propanoic acid with *n* = 6 samples per group. **(C)** The serum concentration of Butyric acid with *n* = 6 samples per group. **(D)** The heatmap represents the mRNA expression levels of pro-inflammatory markers in primary mice AMs with *n* = 6 samples per group. Mice primary AMs were pretreated with butyrate (0.05 and 0.2 mM) or propionate (0.5 and 1 mM) for 20 h and then were treated with LPS (10 ng/ml) for 4 h. The relative expression data were normalized by using log scale and column scale, and the heatmap was drawn in TBtools software. The average expression levels were labeled in each cell. **(E)** The relative p-p65 expression level in AMs was presented by the ratio of p-p65 to p65 with *n* = 3 samples per group. High expression of p-p65 represents the activation of NF-κB signals in lung. Statistical significance was determined by one-way ANOVA using GraphPad Prism 8 software. ^**^p < 0.01, ^***^p < 0.001, ^****^p < 0.0001.

Short-chain fatty acids are important metabolite of intestinal microbiome that have been demonstrated to have anti-inflammation effects. To confirm if the anti-inflammation effects of APS was associated with SCFAs, the SCFAs concentration in serum were measured by GC–MS. The results showed that acetic acid, propanoic acid and butyric acid were significantly increased by APS treatment (Figures 5A–C). Next, we isolated mice primary AMs to further observe the anti-inflammation effects of butyrate and propionate. Cells were pretreated with butyrate or propionate and then were exposed to LPS. The qPCR results showed that butyrate and propionate pre-treatment significantly reduced the mRNA levels of pro-inflammatory cytokines, and reduced the expression level of Cd86, the marker of M1 phenotype of macrophages (Figure 5D). These proved the anti-inflammation effects of butyrate and propionate. We further measured the activation of NFκB signaling. Western blot results showed that the ratio of p-p65/p65 was significantly increased following LPS treatment, while butyrate and propionate pre-treatment inhibited the increase of p-p65 induced by LPS, suggesting the inhibition of NFκB signaling (Figure 5E).

## Discussion

The excessive inflammatory response induced by LPS is considered as the main reason for the injury of lung (Weng et al., 2011), and therefore is the primary target to prevent the disease. Several natural products have been proven to be capable of preventing the onset of lung injury. As one of the most extensively studied natural product, APS has multiple bioactivities, nonetheless, its preventive effects on inflammatory lung injury are still unknown.

In this study, we evaluated the preventive effects of APS on inflammatory lung injury induced by LPS. The monosaccharides composition of APS was firstly characterized, as it would affect the microbiota composition and metabolism. Then mice were pretreated with purified APS for 14 days before exposed to LPS. APS did not affect the body weight of mice, indicating the safety of the dosage. We successfully established the inflammatory lung injury mice, which were manifested damaged alveolar structure, lung edema, and the infiltration of neutrophils. All these pathological changes were ameliorated by APS pre-treatment, proved the efficacy of APS in protecting host from inflammatory lung injury. APS pre-treatment also greatly suppressed the inflammatory response in lung tissue and inhibited NFκB signaling pathway by which immune cells produce inflammatory cytokines (Lu et al., 2008). ICAM-1 can be upregulated in inflammation and mediates neutrophils adhesion to endothelial cells (Wiesolek et al., 2020). Neutrophils are one of the main cell types that produce inflammatory cytokines. APS pre-treatment significantly inhibited the infiltration of neutrophils, which was probably due to the decreased expression of *Icam-1* after APS treatment. Moreover, APS also reduced the relative expression of *Cd86* while increased the relative expression of *Mrc1*, suggested the transition of lung-resident macrophages from pro-inflammatory phenotype (M1) to anti-inflammatory phenotype (M2), and thereby reduced the production of pro-inflammatory cytokines (Hussell and Bell, 2014). Previous studies have demonstrated the anti-inflammatory effects of APS *in vitro* and *in vivo* (He et al., 2012; Lv et al., 2017), our data provided additional evidences for the protective effects of APS on lung inflammation.

Although the mechanisms are largely unknown, increasing evidence confirmed the intimate relationship between intestinal microbiota and the lung immunity (Samuelson et al., 2015). The alteration of intestinal microbiota may have profound effects on lung diseases. Non-starch polysaccharides cannot be fully digested and assimilated by host and thus become the major nutrients reaching the intestinal microbiota (Porter and Martens, 2017). The beneficial effects of herbal polysaccharides on host immune response have been shown to relate to the alteration of intestinal microbiota (Tang et al., 2019). Therefore, we speculated that the effects of APS on the inflammatory lung injury was related to the alteration of intestinal microbiota. The mice colons were collected for 16S rRNA sequencing and the results showed that APS pre-treatment increased the abundance of Firmicutes and decreased abundance of Bacteroidetes at phylum level. These two are the most important bacterial phyla in gastrointestinal tract and greatly affect the host health. Supplementing members from Firmicutes or increasing the abundance of Firmicutes through dietary fiber have been demonstrated to improve the host health condition (Sun et al., 2022). Nonetheless, the effects of microbiota on host are complex. Increased Firmicutes can also produce LPS and deoxycholic acid and increase the risk of inflammation in liver, while several species in Bacteroidetes are dominant beneficial bacteria, which provides nutrition and vitamin for host (Yoshimoto et al., 2013; Zafar and Saier, 2021).

At genus level, we found that APS pre-treatment increased the abundance of *Oscillospira*, *Akkermansia*, and *Coprococcus*. Both *Oscillospira* and *Coprococcus* belong to Firmicutes and are implicated in many diseases. The abundance of *Oscillospira* can be significantly reduced in inflammatory disease such as IBD and nonalcoholic steatohepatitis (Zhu et al., 2013; Walters et al., 2014), indicated that high abundance of *Oscillospira* is important for suppressing inflammatory response. The only positive relationship between *Oscillospira* and disease phenotype was observed in patients with gallstones, showing increased *Oscillospira* abundance (Keren et al., 2015). More importantly, *Oscillospira* has been proven to produce SCFAs dominated by butyrate, which make it the promising candidate for the next-generation probiotics (Yang et al., 2021). Despite the relative low abundance, *Akkermansia* and *Coprococcus* were demonstrated to play important role in maintaining gut homeostasis and inhibiting inflammation (Everard et al., 2013; Gevers et al., 2014). Moreover, both *Akkermansia* and *Coprococcus* can also produce SCFAs including butyrate and propionate (Louis et al., 2014). Our data showed that LPS treatment increased the abundance of *Prevotella*, a large genus with high species diversity but as well as many opportunistic bacteria, while APS pre-treatment reversed this change. *Prevotella* positively affect host metabolism, but studies found that *Prevotella* colonization promoted the dextran sulphate sodium-induced colitis, it was also associated with new-onset rheumatoid arthritis, implying its pro-inflammatory effects (Larsen, 2017). Suppressing the excessive *Prevotella* abundance may be helpful for reducing the inflammatory response of the host.

Polysaccharides reaching the intestine can be fermented by the microbiota, and the chemical energy in carbon is converted into ATP that is used by cells in intestine. The major products of this fermentation are SCFAs including butyrate, acetate, and propionate (Cummings et al., 1987). Although SCFAs are usually used locally by enterocytes, they can be transported across the intestine epithelium into the blood circulation and act in lungs (Enaud et al., 2020). As mentioned above, species in *Oscillospira*, *Akkermansia*, and *Coprococcus* can utilize plant material to produce SCFAs, and as expected, we found that the concentrations of acetate, butyrate, and propionate in serum were significantly increased after APS treatment. Studies have confirmed that SCFAs regulate lung immunity. Increased SCFAs alleviated lung injury induced by *K. pneumoniae* by suppressing macrophages mediated inflammatory response, while the reduction of SCFAs impaired the function of alveolar macrophages and promote the resulting lung superinfection (Sencio et al., 2020; Ting et al., 2020). Our data also suggested that butyrate and propionate, two major SCFAs with immunoregulation bioactivities, markedly inhibited the activation of NFκB signaling on mice AMs, and the consequent production of pro-inflammatory cytokines. These effects would further contribute to the alleviation of lung injury induced by LPS.

Altogether, this study demonstrated the beneficial effects of APS on alleviating inflammatory lung injury. APS pre-treatment altered the composition of intestinal microbiota, increased the abundance of SCFAs-producing bacteria, as well as the serum concentration of butyrate and propionate. These effects constituted the underlying mechanisms by which APS pre-treatment alleviated LPS-induced inflammatory lung injury.

## Data availability statement

The datasets presented in this study can be found in online repositories. The names of the repository/repositories and accession number(s) can be found in the article/supplementary material.

## Ethics statement

The animal study was reviewed and approved by Ethics Committee of Huazhong Agricultural University.

## Author contributions

MD and YD participated in the design of this study, and revised the manuscript. KM carried out the experiments and data analysis and drafted the manuscript. SZ, NM, SN, and QL provided necessary assistance for samples collection and data analysis. All authors contributed to the article and approved the submitted version.

## Funding

The project was supported by China Postdoctoral Science Foundation (Grant No. 2021M701350) and National Natural Science Foundation of China (Grant Nos. 32072938 and 32172930).

## Conflict of interest

The authors declare that the research was conducted in the absence of any commercial or financial relationships that could be construed as a potential conflict of interest.

## Publisher’s note

All claims expressed in this article are solely those of the authors and do not necessarily represent those of their affiliated organizations, or those of the publisher, the editors and the reviewers. Any product that may be evaluated in this article, or claim that may be made by its manufacturer, is not guaranteed or endorsed by the publisher.
